# Evaluation of a microfluidic chip system for preparation of bacterial DNA from swabs, air, and surface water samples

**DOI:** 10.1016/j.biologicals.2016.06.013

**Published:** 2016-11

**Authors:** Sandra Julich, Helmut Hotzel, Claudia Gärtner, Daniel Trouchet, Marwa Fawzy El Metwaly Ahmed, Nicole Kemper, Herbert Tomaso

**Affiliations:** a Friedrich-Loeffler-Institut, Federal Research Institute for Animal Health, Institute of Bacterial Infections and Zoonoses, Naumburger Straße 96a, 07743 Jena, Germany; b microfluidic ChipShop, Stockholmer Straße 20, 07747 Jena, Germany; c Bertin Technologies, 10 Avenue André Marie Ampére, 78180 Montigny-le-Bretonneux, France; d Mansoura University, Faculty of Veterinary Medicine, Department of Animal Hygiene and Zoonoses, 60 El Gomhoria Street, 35516 Mansoura, Egypt; e University of Veterinary Medicine Hannover, Foundation, Institute of Animal Hygiene, Animal Welfare and Farm Animal Behaviour, Bischofsholer Damm 15, 30173 Hannover, Germany

**Keywords:** Microfluidic chips, *Francisella tularensis*, *Bacillus thuringiensis*, DNA preparation, Environmental samples, *Campylobacter*

## Abstract

The detection of bacterial pathogens from complex sample matrices by PCR requires efficient DNA extraction. In this study, a protocol for extraction and purification of DNA from swabs, air, and water samples using a microfluidic chip system was established. The optimized protocol includes a combination of thermal, chemical and enzymatic lysis followed by chip-based DNA purification using magnetic particles. The procedure was tested using Gram-positive *Bacillus thuringiensis* Berliner var. *kurstaki* as a model organism for *Bacillus anthracis* and the attenuated live vaccine strain of *Francisella tularensis* subsp. *holarctica* as Gram-negative bacterium. The detection limits corresponded to 10^3^ genome equivalents per milliliter (GE/ml) for surface water samples spiked with *F. tularensis* and 10^2^ GE/ml for *B. thuringiensis*. In air, 10 GE of *F. tularensis* per 10 L and 1 GE of *B. thuringiensis* per 10 L were detectable. For swab samples obtained from artificially contaminated surfaces the detection limits were 4 × 10^3^ GE/cm^2^ for *F. tularensis* and 4 × 10^2^ GE/cm^2^ for *B. thuringiensis*. Suitability of the chip-assisted procedure for DNA preparation of real samples was demonstrated using livestock samples. The presence of thermophilic *Campylobacter* spp. DNA could be confirmed in air samples collected on pig and broiler farms.

## Highlights

• A microfluidic chip system for magnetic bead-based DNA preparation was evaluated.
• Bacterial DNA was recovered from swabs, air, and surface water.
• A universal protocol was used for Gram-positive and Gram-negative bacteria.
• 10 GE of *F. tularensis* and 1 GE of *B. thuringiensis* per 10 l air were detectable.
• Thermophilic *Campylobacter* DNA was detected in air samples of pig and broiler farms.

## 1 Introduction

Cultivation of bacteria originating from environmental samples is often difficult and time consuming. Real-time PCR assays allow a rapid and specific detection of bacteria such as *Campylobacter jejuni* [1] and *Francisella tularensis* [2], [3], [4] without cultivation, but components in environmental samples can act as inhibitors for PCR assays, like e.g. urban dust in air samples [5]. Therefore, the target DNA has to be extracted and purified prior to PCR. The procedure includes disrupting bacterial cell walls, collecting nucleic acids and removal of all other components of the bacterium and matrix. Thermal lysis and mechanical procedures like grinding or sonication as well as chemical lysis with lysozyme and chaotropic salt buffers are common methods for cell disruption. PCR inhibitors are removed by washing steps after DNA binding. Most common DNA purification processes rely on adsorption of DNA onto a solid surface, either via hydrogen bonding to silica or via electrostatic interactions. Particles with special surfaces and a paramagnetic core can be retained with a magnetic device. These particle and matrix associated approaches are more suitable for miniaturization than the traditional chloroform-phenol extraction [6]. Various chip gadgets have been developed for preparation of DNA from bacteria [7], [8], [9], [10], [11]. Silica beads [7], [8] with superparamagnetic iron oxide cores [9] are also very common for on-chip application. Microfluidic lab-on-a-chip devices need only small volumes of reagents and samples. They are portable and can be used for fast analysis at the point-of-need [12]. The number of microfluidic devices which are actually used for preparation of DNA from environmental samples is still very low and their suitability was demonstrated only for specific kinds of samples like swabs [13] or aerosols [8]. A microfluidic method for preparation of DNA from Gram-negative or Gram-positive bacteria in different matrices is a prerequisite for PCR and could be an innovative tool for diagnostic purposes.

*Bacillus* (*B.*) *anthracis* and *Francisella* (*F.*) *tularensis* are zoonotic bacteria that cause fatal diseases in animals and humans and are endemic in many areas around the world. Both bacterial species are classified as category A bioterrorism agents by the US Centers for Disease Control and Prevention (CDC, USA) (http://www.bt.cdc.gov/agent/agentlist-category.asp). *Bacillus anthracis*, the causative agent of anthrax, is a Gram-positive bacterium that forms spores which can persist in the environment for decades [14]. Typically, animals become infected by direct contact with soil containing spores [15], but also grazing, contaminated food or vectors are possible routes of infection [16]. Mortality is highest, if anthrax is acquired by inhalation, but also gastrointestinal infections can be fatal [17]. In September and October 2001 letters containing *B. anthracis* spores were mailed to several US locations as bioterrorist attacks [18], [19]. The closely related bacterium *Bacillus thuringiensis* [20] is a common plant protection agent that is pathogenic for insects, but non-pathogenic for vertebrates including humans. Therefore, *B. thuringiensis* has frequently been suggested as substitute of *B. anthracis* for field exercises. *F. tularensis*, the causative agent of tularemia, is widespread throughout the northern hemisphere and regarded as a potential biological warfare agent [21]. Airborne transmission can occur during processing of agricultural products or handling of infected animals [22]. The organism is also known to persist in water or mud for weeks to months. *Francisella*-specific DNA has been identified from natural water sources [23] or soil [24] and *F. tularensis* contaminated water has caused severe outbreaks in humans [25]. Thermophilic *Campylobacter* (*C.*) species, especially *C. jejuni* and *Campylobacter coli*, are important zoonotic agents that cause gastrointestinal infections after consumption of contaminated food [26], [27]. *C. jejuni* specific DNA has already been detected by PCR in air samples in the surroundings of hen flocks [28].

Here, we present an efficient magnetic bead-based DNA preparation method using a microfluidic chip system, which was used for the preparation of bacterial DNA from environmental samples such as swabs, air, and surface water as a prerequisite for real-time PCR assays. The system was evaluated using the model bacterium *B. thuringiensis* and a live vaccine strain of *F. tularensis* what was beneficial for security and safety reasons. For a realistic evaluation the system was challenged with environmental air samples from broiler chicken and pig livestock with PCR assays targeting *Campylobacter* species.

## 2 Materials and methods

### 2.1 Bacterial strains and environmental samples

The live vaccine strain (LVS) *F. tularensis* subsp. *holarctica* (ATCC 29684) was obtained from the Bundeswehr Institute of Microbiology (Munich, Germany). It was used instead of the highly pathogenic subspecies *F. tularensis* subsp. *tularensis*. Cultivation was carried out on 10% sheep blood/cystein heart agar plates at 37 °C in 5% CO_2_ atmosphere. Colony material was suspended in phosphate-buffered saline (PBS).

A commercial formulation of the spore forming *B. thuringiensis* Berliner var. *kurstaki* (Dipel ES, Cheminova Deutschland, Stade, Germany) was used as a non-pathogenic substitute of *B. anthracis*.

Quantitative results were shown as the number of genome equivalents (GE) per ml or calculated to corresponding concentrations per liter air or per surface unit (cm^2^). Water and air samples were suspended in PBS and spiked with bacteria suspensions to final concentrations between 10 and 10^8^ GE/ml. Surface water was collected in October 2014 from the river Saale near Jena, Germany. A portable instrument (Coriolis μ, Bertin Technologies, Montigny-le-Bretonneux, France) was used for collecting air samples. Samples of ambient air were collected between January and April 2014 at the area of the Friedrich-Loeffler-Institut in Jena, Germany. Air temperature, air pressure, wind speed, and wind direction were measured using a standard weather station (PCE-FWS 20, PCE Deutschland, Meschede, Germany). Corresponding weather data including the regional pollen count are provided as supplementary data in Table SI. The air sampler operated for 60 min at a flow rate of 300 l/min to deposit airborne particles in a cone. The collected particles from each cone were suspended in 13 ml PBS and pooled afterwards. For detection of different *Campylobacter* species air samples were taken in a broiler chicken and a pig farm in Lower Saxony, Germany. Sampling in the stables was carried out 8 times each at two different levels above the ground for 3 min using a flow rate of 300 l/min. 15 ml PBS containing 0.01% Tween 20 and 10 mM ascorbic acid were used for solubilization. Air samples were taken at 150 cm in both stables and also at 30 cm for broilers and 50–60 cm above the ground for pigs. Swab samples were prepared from sterile glass plates which had been coated with serial dilutions of bacterial suspension (between 10 and 10^8^ GE/ml) on an area of 5 × 5 cm^2^. Rayon-tipped plastic swabs (Copan Italia, Brescia, Italy) were moistened with 50 μl PBS before the sample was taken. The swabs were rotated and rubbed in a zig-zag pattern over the selected air-dried area. Swab tips and 2 ml PBS were transferred to test tubes and stirred thoroughly for 30 s (REAX 2000 Shaker, Heidolph International, Schwabach, Germany). The swab tips were transferred into clean reaction vessels and residual liquid was extracted by centrifugation for 1 min at 12,000 × *g* (Eppendorf miniSpin, Eppendorf, Hamburg, Germany).

All spiked and contaminated sample materials were prepared in quadruplicates. Each of the eight real air samples and all no-template control samples were prepared in duplicates. All eluates were analyzed in duplicates. An overview about the sample preparation procedure used for different spiked environmental samples is shown in Fig. S1.

### 2.2 Microfluidic chip system

**Fig. 1:**
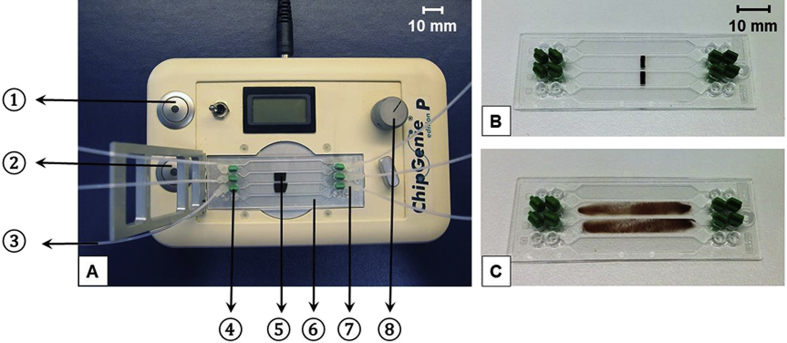
Microfluidic chip system for purification of DNA. A instrument with inserted chip; ➀ on/off switch for heating unit; ➁ on/off switch for magnet motion; ➂ flexible tubes; ➃ mini-Luer plugs; ➄ magnetic particles; ➅ microfluidic chip including four separate cavities; ➆ mini-Luer connectors; ➇ temperature setting; B chip device including magnetic particles arranged in stripes; C chip device including mixed magnetic particles.

Disposable microfluidic chips (microfluidic ChipShop, Jena, Germany) in microscope slide format (height: 1.5 mm, width: 75.5 mm, length: 25.5 mm) made of cyclic olefin polymer (COP) were used for purification of DNA (Fig. 1B, Fig. 1C). Each chip contained four rhombic cavities. Each cavity was connected with mini-Luer inlet and outlet ports. The volume of each chamber was 100 μl. Liquid reagents were sucked through polytetrafluoroethylene (PTFE) tubes (Bohlender, Grünsfeld, Germany) connected with Tygon Tubes (ST R-3607, IDEX Health Science, Wertheim, Germany) into the chip cavities using a peristaltic pump (Ismatec Reglo digital, IDEX Health Science, Wertheim, Germany). The Tygon tubes had an inner diameter of 510 μm and a wall thickness of 910 μm. The PTFE tubes had an inner diameter of 500 μm and a wall thickness of 250 μm. The tubes were attached to the outlet port by mini-Luer connectors. Remaining ports were sealed with mini-Luer plugs (microfluidic ChipShop) to avoid evaporation of liquids inside the cavities. The apparatus (ChipGenie edition P, microfluidic ChipShop) used in conjunction with the microfluidic chip devices included a temperature control unit as well as a movable magnet for particle mixing (Fig. 1A).

### 2.3 DNA preparation methods

Different procedures for chip-assisted preparation of DNA were pre-tested in comparison to a standard off-chip method using the High Pure PCR Template Preparation Kit (Roche Diagnostics, Mannheim, Germany) according to the manufacturer's instructions. Additionally, untreated samples were analyzed as reference. The performance of the different DNA extraction and on-chip DNA purification procedures (Table 1) was evaluated using bacteria suspensions diluted in PBS and spiked environmental samples. The final bacteria concentration corresponded to 10^9^ GE/ml. Two ml of sample solution were used for each on-chip DNA preparation process and 100 μl of eluate were obtained after the process was finished. For chip-assisted DNA preparation procedures, different combinations of the following steps were tested.

#### 2.3.1 Lysis

##### 2.3.1.1 Heat treatment

Thermal lysis was carried out at 95 °C for 10 min (Thermomixer compact, Eppendorf, Hamburg, Germany).

##### 2.3.1.2 Enzymatic lysis with lysozyme

30 μl of 50 mg/ml lysozyme (Carl Roth, Karlsruhe, Germany) in 10 mM Tris–HCl buffer (pH 8.0, Carl Roth) were added to the sample and incubation was carried out at 37 °C for 15 min (Thermomixer compact, Eppendorf).

##### 2.3.1.3 Chemical lysis

One ml lysis buffer (pH 4.0) containing 6 mM guanidinium-HCl (Carl Roth), 10 mM Tris–HCl, 10 mM urea (Merck, Darmstadt, Germany) and 30% Triton-X-100 (Serva Electrophoresis, Heidelberg, Germany) was added to the sample solution. Furthermore, one ml isopropanol (Berkel AHK Alkoholhandel, Berlin, Germany) was added.

##### 2.3.1.4 Enzymatic lysis with proteinase K

50 μl of 10 mg/ml proteinase K (Carl Roth) were added and the samples were incubated at 70 °C for 10 min (Thermomixer compact, Eppendorf).

#### 2.3.2 Magnetic particle application

##### 2.3.2.1 Magnetic particles as stripe

Magnetic particles (6 mg, 80% with a diameter between 5 and 10 μm) were pre-loaded into the microfluidic chip and concentrated as a stripe located in the middle of the cavity (Fig. 1B). Then, the sample solution was pumped continuously through the cavity using a flow rate of 100 μl/min. This was done at 55 °C except for procedure 2, in which room temperature was used.

##### 2.3.2.2 Magnetic particles mixed with sample

Before loading the chip, the lysate was mixed with the magnetic particles (6 mg) and incubated for 15 min at 55 °C with a mixing speed of 500 rpm (Thermomixer compact, Eppendorf). This mixture was pumped through the chip cavities to collect magnetic particles in the middle using the integrated magnet.

#### 2.3.3 Washing and elution

Washing was carried out at room temperature by pipetting the liquids into the cavities and closing all ports with mini-Luer plugs. During washing and elution the particles were continuously moved inside the cavity by magnet motion (Fig. 1C). The magnet was placed stationary below the middle of the chip to retain the particles inside of the cavity when washing buffer and eluate were removed. After washing twice with 100 μl washing buffer (microfluidic ChipShop) for 30 s, another washing step was carried out for 15 min with 100 μl water. After each washing step liquids were removed from the cavities. Elution was carried out with 100 μl TE buffer (Carl Roth) for 5 min at 55 °C.

### 2.4 Real-time PCR

In order to generate a standard curve for quantification, DNA was extracted from bacterial colonies using a standard phenol-chloroform DNA extraction protocol [6]. The DNA concentration was measured using a NanoDrop 2000c spectrophotometer (NanoDrop products, Wilmington, DE, USA). Serial dilutions of DNA in PCR-grade water were prepared with concentrations ranging from 1 ng to 10 fg per reaction for all real-time PCR assays. The details of all PCR assays are given in Table 2. Each PCR mixture had a total volume of 25 μl and contained 1× LightCycler 480 Probes Master (Roche Diagnostics GmbH), 400 nM primers (TIB MOLBIOL Syntheselabor, Berlin, Germany) and 100 nM probe labeled with carboxyfluorescein (FAM) and tetramethylbenzidine (TAMRA). Eluates were tested in quadruplicate measurements with 10 μl in each PCR mixture. PCR conditions are given in Table 2. Water was used as negative control. Quantitative real-time PCR assays were performed with a LightCycler 480 Instrument II (Roche Diagnostics) that was used with excitation at 465 nm and detection at 510 nm. Absolute Quantification Analysis and the Fit Points method were used as provided by the LightCycler 480 software (Roche Diagnostics) for determination of the cycle threshold (C_T_) values. Electrophoresis on 1.5% agarose gel (Agarose GTQ, Carl Roth) was used to verify the expected size of PCR products. After staining with GelRed (Biotium, Hayward, CA, USA) PCR products were visualized under UV light.

## 3 Results

### 3.1 Selection of optimal procedure for DNA extraction and purification using the microfluidic chip system

Different methods for chip-assisted DNA preparation (Table 1) of samples spiked with bacteria in concentration of 10^9^ GE/ml were compared with those obtained using a reference kit and detection without sample preparation. *B. thuringiensis* and *F. tularensis* were either added to PBS or air samples collected with PBS. Procedure 4 proved to be most efficient for the detection of both agents. The protocol included a combination of thermal, enzymatic and chemical lysis methods with DNA adherence to continuously mixed magnetic particles for purification (Table 1). Table 3 obtained C_T_ values and total processing time for different DNA preparation methods. Bacteria were suspended with pure PBS or air samples collected with PBS. The best (lowest) C_T_ values for air samples spiked with *F. tularensis* were achieved using procedure 4. For *B. thuringiensis* the lowest C_T_ values were achieved using procedure 2, which requires only the shortest processing time. However, this procedure is less efficient for preparation of samples spiked with *F. tularensis*. Although procedure 4 required the longest processing time, it was the most promising approach for low concentrations of bacteria.

### 3.2 Chip-assisted DNA purification for bacteria detection from spiked environmental samples

The chip-assisted sample preparation procedure 4 was evaluated for its ability to recover *F. tularensis* and *B. thuringiensis* DNA from different artificially contaminated environmental samples. For this purpose, final concentrations between 10 GE/ml and 10^8^ GE/ml were prepared with surface water and air samples collected in PBS. Bacteria suspensions with these concentration levels were also applied on glass plates that were used to test swab sampling. In general, both bacteria species were successfully detected in all four sample matrices (Table 4). For air and water samples lowest detection limits were achieved using the chip-assisted sample preparation procedure 4. Detection limits of 10^2^ GE/ml for *B. thuringiensis* and 10^3^ GE/ml for *F. tularensis* were achieved for both kinds of samples. The detected concentrations for air samples corresponded to 10 GE per 10 l air for *F. tularensis* and 1 GE per 10 l air for *B. thuringiensis*. Bacterial concentrations of 10^5^ GE/ml *B. thuringiensis* and 10^6^ GE/ml *F. tularensis* could be detected in samples originating from surface water. Concentrations of 4 × 10^3^ GE/cm^2^ for *F. tularensis* and 4 × 10^2^ GE/cm^2^ for *B. thuringiensis* were detectable using swab samples.

### 3.3 Detection of thermophilic *Campylobacter* species from environmental air samples

The chip-assisted sample preparation procedure proved to be suitable for detection of different *Campylobacter* species from air samples collected in broiler chicken and pig stables. In both stables DNA of three thermophilic *Campylobacter* species were detected (Table 5). *C. coli* was found in 23 out of 32 air samples from a chicken stable. The highest concentration of *Campylobacter* (mean value: 184 GE per 10 l air) was detected when the air samples were collected 30 cm above the ground. In these air samples also 100 GE of *Campylobacter lari* per 10 l air were detected. In a pig stable 16 out of 32 samples were positive for *C. lari* and 25 out of 32 samples were positive for *C. coli*. In this setting, the highest concentrations of *Campylobacter* were found 150 cm above the ground. Low concentrations of *C. jejuni* were detected sporadically. Without sample treatment, none of the samples yields positive PCR results for any of the *Campylobacter* species (data not shown).

## 4 Discussion

The aim of this study was to optimize a protocol for DNA preparation and purification from different matrices using a microfluidic chip device. With the selected protocol an increased DNA yield was obtained especially for air samples contaminated with *F. tularensis* and *B. thuringiensis* compared to samples prepared with the reference kit or without any preparation. For the first time, different environmental samples were investigated using a universal protocol with a microfluidic chip system to detect model organisms for potential biological agents.

For DNA purification from complex sample matrices it is essential to combine thermal, enzymatic and mechanical steps as included in procedure 4, although the processing time is longer than for other procedures. A combination of different lysis methods can also improve the recovery of DNA from Gram-positive, spore-forming bacteria species. The major advantage of this protocol is its universal applicability for preparation of DNA from different environmental samples contaminated with unknown bacterial targets. Targets can comprise Gram-negative and Gram-positive bacteria. Increased DNA concentrations achieved for the eluates prepared with this protocol is another advantage and can be explained also by an enrichment effect. Since the volumetric capacity of the chip cavity was just 100 μl and a sequential procedure was not possible most preparation steps were performed in a suitable reaction vessel off-chip. The presented chip system was exclusively used for collection of magnetic particles with captured DNA as well as very efficient performance of washing and elution. Zhang et al. [29] described a microfluidic chip system that increased DNA concentration of the samples. However, this system was not challenged with environmental samples.

The achieved detection limits are suitable for the intended purpose. However, further improvement is required.

In order to enable a point-of-care application of the system, further automation would be essential to avoid manual handling steps.

The major advantage of magnetic beads in comparison to other materials for reversible attachment of nucleic acids is their mobility that allows for different applications including concentration to a stripe or continuous movement that favors careful washing and elution. Moreover, they can easily be filled into the cavities by pipetting of particle solution.

Procedure 2 was carried out using the microfluidic chip system by placing a stripe of concentrated magnetic particles in the middle of the cavities. This protocol requires the shortest processing time. It can be assumed that the concentrated particles act as a mechanical filter in this case. With the particles used in our study the obtained interspaces are larger than 1.2 μm. *B. thuringiensis* cells have an average size between 0.5 μm × 1.2 μm and 2.5 μm × 10 μm, which results in retention of a large amount of cells on the particle stripe. *F. tularensis* cells are much smaller (0.2 μm × 0.2 μm × 0.7 μm) and can therefore slip through the interspaces. This could explain the high DNA yield achieved for *B. thuringiensis* using procedure 2, while DNA yield from *F. tularensis* was markedly lower. Application of smaller magnetic particles that can result in interspaces of smaller size could enable preparation also of tiny bacteria.

A commercial test kit for DNA extraction and purification was used as reference method. The reference kit enables fast processing of samples within 32 min, but is recommended for 10^9^ cells according to the instructions of the manufacturer. The selected method for DNA extraction and purification using a microfluidic chip system allows for species identification including subsequent real-time PCR within 3 h and 15 min.

The achieved detection limits seem to be adequate for surveillance of bacteria, especially for air samples. Low detection limits are important especially for highly pathogenic species that can lead to serious infections due to inhalation of only a few bacteria. 8000–50,000 spores of *B. anthracis* [30] and only 10–50 bacteria of *F. tularensis* [31] can cause a pulmonary infection. Taking into consideration the average respiratory volume of an adult with approximately 8 l/min and the achieved detection limit in air, the method could be integrated in an early warning system against infectious bacteria and might be also suitable for point-of-care-testing in critical areas. However, according to the producer of the air-sampler used in this study the efficiency of air-sampling itself is approximately 70% which further underlines the importance of efficient DNA preparation.

For surface water samples taken from a river artificially contaminated concentrations to 10^3^ GE/ml for *F. tularensis* and 10^2^ GE/ml for *B. thuringiensis* could be detected. Therefore, the limit of detection should be sufficient to detect relevant concentrations of bacteria that might cause infections via the intestinal route.

Results for swab detection in the literature are often based on DNA extraction of swabs soaked with bacteria suspension, which is not a very realistic scenario. Walker et al. [32] were able to detect *F. tularensis* with an amount of only 100 colony forming units (CFU)/swab using off-chip methods based on magnetic beads and spin columns filled with silica fleece. Sixty one CFU of *Staphylococcus aureus* per swab could be detected inside of a chip cavity packed with glass beads by Hwang et al. [7] Recovery of pathogens from surfaces is quite different, because it is impossible to collect all bacteria from surfaces swabs as was shown by Martinon et al. [13] who detected 10^6^ CFU/cm^2^ *Escherichia coli*, *S. aureus*, and *Listeria monocytogenes* with a standard laboratory method.

It was demonstrated that the described system is suitable for testing environmental samples. DNA of different *Campylobacter* species was detected in air samples originated from stables, while viable cells of this microaerophilic species cannot be detected in air [28]. Bacteria identification from environmental samples is challenging due to the content of various PCR inhibitors. Because real environmental conditions are highly variable the study was performed mainly with spiked samples. The results demonstrate that especially for low DNA concentrations not all replicates could be detected.

## 5 Conclusion

In this study, different sample preparation methods were compared using a microfluidic chip system. A combination of thermal, chemical, and enzymatic lysis with magnetic bead-based DNA purification resulted in the best performance for *B. thuringiensis*, which was used as substitute for *B. anthracis* and *F. tularensis* vaccine strain. The bacteria could be detected in environmental samples with low detection limits. The suitability of this chip-assisted sample preparation procedure was proven for detection of *Campylobacter* species in air samples from animal farms. There is a low novelty level for the presented chip system from technical point of view, but the universal character of the presented application might be interesting for analyzing environmental samples. Future developments will focus on the combination of microfluidic sample preparation and target detection procedures [33] to create an all-in-one-systems.

## Figures and Tables

**Table 1:** Detailed protocol arrangement of the different chip-assisted DNA preparation procedures.

	Procedure 1	Procedure 2	Procedure 3	Procedure 4
Magnetic particle	–	Stripe	–	–
Heat treatment	95 °C, 10 min			
Enzymatic lysis	–	–	Lysozyme	
Chemical lysis	–	–	Lysis buffer, Isopropanol	
Enzymatic lysis	–	–	Proteinase K	
Magnetic particle	Stripe	–	Stripe	Mixed
Washing	2× Washing buffer, 1× Water			
Elution	100 μl TE buffer			

**Table 2:** PCR assays applied for specific detection of *F. tularensis*, *B. thuringiensis*, *C. jejuni*, *C. coli*, and *C. lari*. With all PCR assays an initial denaturation step of 95 °C for 10 min and a total number of 45 PCR cycles was performed.

Specie	fg DNA per GE	Target gene	Reference	Primer and probe sequences (5′→3′)	Thermocycling protocol
*F. tularensis*	1.95	*fopA*	[3]	Forward: TTGGGCAAATCTAGCAGGTCA	15 s 95 °C, 60 s 60 °C
				Reverse: ATCTGTAGTCAACACTTGCTTGAACA	
				Probe: FAM-AAGACCACCACCAACATCCCAAGCA-TAMRA	
*B. thuringiensis*	5.67	*cryT*	[34]	Forward: ATGGCTTCTCCTGTAGGGCCGCT	15 s 95 °C, 60 s 60 °C
				Reverse: GCTGCATTTCCCATAGTTCCA	
				Probe: FAM-CCAGAATTCACTTTTCCCGCT-TAMRA	
*C. jejuni*	1.68	*mapA*	[1]	Forward: CTGGTGGTTTTGAAGCAAAGATT	15 s 95 °C, 60 s 60 °C
				Reverse: CAATACCAGTGTCTAAAGTGCGTTTAT	
				Probe: FAM-TTGAATTCCAACATCGCTAATGTATAAAAGCCCT-TAMRA	
*C. coli*	1.70	*ceuE*	[1]	Forward: AAGCTCTTATTGTTCTAACCAATTCTAACA	15 s 95 °C, 60 s 60 °C
				Reverse: TCATCCACAGCATTGATTCCTAA	
				Probe: FAM-TTGGACCTCAATCTCGCTTTGGAATCATT-TAMRA	
*C. lari*	1.57	*gyr*	[35]	Forward: CAGCTATACCACTTGATCCATTAAG	30 s 94 °C, 45 s 55 °C, 90 s 72 °C
				Reverse: GATAAAGATACGGTTGATTTGTACC	
				Probe: FAM-TTATGATGATTCTATGAGTGACCTGATG-TAMRA	

**Table 3:** Comparison of DNA preparation methods and direct detection without any sample preparation using 10^9^ GE/ml (n = 8).

Species	Procedure	1	2	3	4	Reference^a^	No preparation
	Processing time (h:min)	0:42	0:38	1:31	1:46	0:32	–
	Spiked matrix	Average C_T_ (mean ± SD)					
*F. tularensis*	Buffer	20.8 ± 0.02	24.8 ± 0.06	27.8 ± 0.09	19.6 ± 0.09	22.5 ± 0.08	22.8 ± 0.19
	Air sample	21.2 ± 0.01	25.0 ± 0.01	20.3 ± 0.10	18.5 ± 0.01	22.3 ± 0.03	25.2 ± 0.26
*B. thuringiensis*	Buffer	20.4 ± 0.43	15.0 ± 0.02	19.0 ± 0.18	17.7 ± 0.42	24.7 ± 0.84	17.9 ± 0.08
	Air sample	20.2 ± 0.04	16.3 ± 0.35	19.2 ± 0.45	17.9 ± 0.09	28.7 ± 0.14	22.2 ± 0.23

a High Pure PCR Template Preparation Kit (Roche Diagnostics GmbH).

**Table 4:** Amplification threshold cycles (mean ± SD) for different concentrations of *F. tularensis* and *B. thuringiensis* in different artificially contaminated samples. The mean value was calculated from positively detected samples which is given in brackets (n = 8).

Sample matrix	Species	Concentration of solubilized samples (GE/ml)							
		10^8^	10^7^	10^6^	10^5^	10^4^	10^3^	10^2^	10
Air	*F. tularensis*	27.1 ± 0.10 (8/8)	31.5 ± 0.02 (8/8)	34.6 ± 0.25 (8/8)	35.9 ± 0.88 (8/8)	37.2 ± 0.70 (6/8)	39.1 ± 0.63 (8/8)	–	–
	*B. thuringiensis*	18.9 ± 0.28 (8/8)	24.7 ± 0.82 (8/8)	28.6 ± 0.18 (4/8)	33.6 ± 0.72 (8/8)	35.9 ± 0.28 (7/8)	36.2 ± 0.21 (7/8)	37.1 ± 0.62 (7/8)	–
Surface water	*F. tularensis*	26.8 ± 0.02 (8/8)	29.3 ± 0.30 (8/8)	33.3 ± 0.44 (8/8)	36.0 ± 0.64 (8/8)	36.6 ± 0.16 (7/8)	37.5 ± 0.34 (3/8)	–	–
	*B. thuringiensis*	21.4 ± 0.32 (8/8)	26.3 ± 0.79 (8/8)	32.5 ± 0.32 (8/8)	34.5 ± 0.07 (8/8)	35.6 ± 0.02 (7/8)	36.7 ± 0.27 (5/8)	38.0 ± 0.18 (4/8)	–
Surface swab	*F. tularensis*	36.1 ± 0.21 (8/8)	37.9 ± 0.66 (4/8)	39.8 ± 0.27 (4/8)	–	–	–	–	–
	*B. thuringiensis*	28.0 ± 0.83 (8/8)	32.4 ± 0.64 (8/8)	35.9 ± 0.99 (8/8)	37.6 ± 0.59 (8/8)	–	–	–	–

**Table 5:** Detection of *Campylobacter* species from air samples in PBS collected at different sampling positions in a broiler chicken and in a pig stable. Samples were prepared with the microfluidic chip system using procedure 4. The number of positively detected samples is given in brackets (n = 16). Concentrations of GE per 10 l air were calculated with real-time quantitative PCR results.

Livestock	Sampling distance from ground (cm)	Mean concentration (GE per 10 l air)		
		*C. jejuni*	*C. coli*	*C. lari*
Broiler chicken (*Gallus gallus domesticus*)	150	30 (1/16)	68 (14/16)	44 (9/16)
	30	2 (1/16)	184 (9/16)	100 (8/16)
Pig (*Sus scrofa domestica*)	150	9 (1/16)	158 (13/16)	176 (9/16)
	50–60	13 (1/16)	21 (12/16)	22 (7/16)
