# Supplementary material for: Evaluation of a microfluidic chip system for preparation of bacterial DNA from swabs, air, and surface water samples
Source: Biologicals. 2016 Nov;44(6):574–80. doi: 10.1016/j.biologicals.2016.06.013 (PMC5119575; doi:10.1016/j.biologicals.2016.06.013)
Supplement: Supplementary file 1 [file mmc1.docx]

**Supplementary material**

**
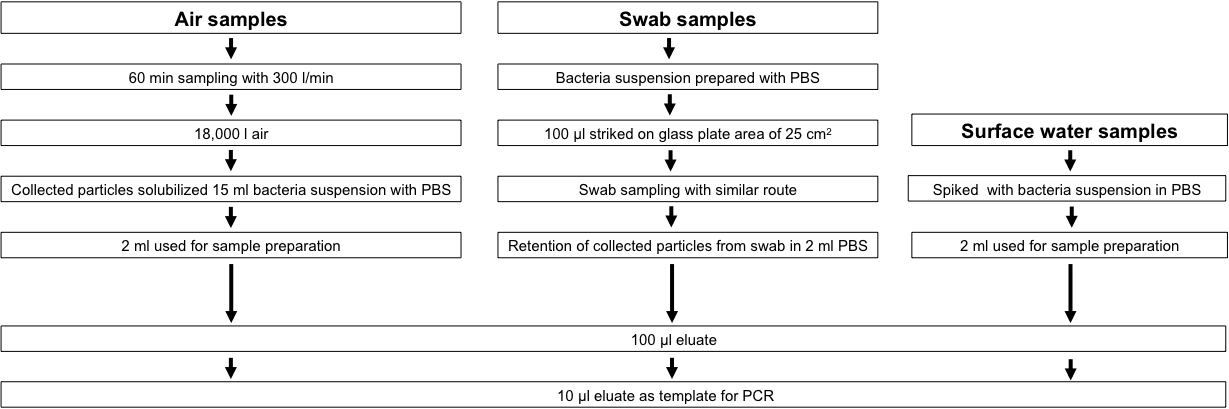
**

Fig. S1. Overview about chip-assisted preparation of different environmental samples spiked with bacteria

Table S1

Weather conditions during air sampling. Generally, air samples were taken unless it rained. The regional pollen count was obtained simultaneously to the sampling (<http://wetter.de/deutschland/pollen-karte-deutschland-c49.html>).

| Use of sample material | Date | Collection time | Temperature [°C] | Wind velocity [km/h] | Wind direction | Air pressure [hPa] | Humidity  [% Realtive humidity] | Pollen count |
| --- | --- | --- | --- | --- | --- | --- | --- | --- |
| Comparison of different sample preparation procedures | 30/01/2014 | Start: 9:00 am | 2.4 | 1.1 | NO | 993.7 | 59 | - |
|  |  | End: 2:30 pm | 2.7 | 6.1 | S | 993.7 | 59 |  |
|  | 31/01/2014 | Start: 9:00 am | -3.5 | 1.1 | O | 992.1 | 78 | - |
|  |  | End: 2:30 pm | 1.4 | 1.1 | W | 992.8 | 72 |  |
|  | 04/02/2014 | Start: 9:00 am | 2.2 | 0.0 | - | 994.5 | 71 | Alder and hazel weak |
|  |  | End: 3:15 pm | 6.5 | 1.1 | SO | 995.8 | 62 |  |
|  | 05/02/2014 | Start: 9:00 am | 0.6 | 1,1 | S | 986.7 | 74 | Alder and hazel weak |
|  |  | End: 11:00 am | 7.4 | 3.6 | SO | 985.7 | 56 |  |
|  | 05/03/2014 | Start: 11:00 am | 10.7 | 5.0 | W | 1001.1 | 58 | Alder and hazel high |
|  |  | End: 2:00 pm | 10.4 | 5.0 | W | 1001.5 | 56 |  |
|  | 06/03/2014 | Start: 7:30 am | 6.4 | 1.1 | SO | 1014.0 | 88 | Alder and hazel high |
|  |  | End: 11:00 am | 7.5 | 1.5 | SO | 1013.8 | 86 |  |
| Validation of the optimal procedure for chip-assisted preparation of air samples | 26/03/2014 | Start: 10:15 am | 8.2 | 1.1 | SW | 998.6 | 66 | Ash, birch, alder and |
|  |  | End: 3:00 pm | 10.5 | 5.0 | O | 997.9 | 46 | hazel weak to moderate |
|  | 27/03/2014 | Start: 9:00 am | 5.6 | 1.1 | SO | 999.4 | 82 | Ash, birch, alder and |
|  |  | End: 2:15 pm | 14.1 | 12.2 | SO | 997.0 | 44 | hazel weak to moderate |
|  | 28/03/2014 | Start: 6:30 am | 1.4 | 1.1 | SO | 1002.9 | 90 | Ash and birch high, alder |
|  |  | End: 1:30 pm | 9.2 | 2.5 | SSO | 1003.8 | 79 | and hazel moderate |
|  | 31/03/2014 | Start: 9:45 am | 7.9 | 1.1 | SO | 998.8 | 85 | Birch and ash high |
|  |  | End: 2:00 pm | 17.5 | 1.1 | S | 997.9 | 52 |  |
|  | 01/04/2014 | Start: 10:00 am | 10.1 | 2.5 | SO | 997.5 | 79 | Birch and ash moderate |
|  |  | End: 3:00 pm | 19.5 | 6.1 | O | 995.8 | 44 |  |
|  | 02/04/2014 | Start: 9:00 am | 12.2 | 1.1 | S | 994.5 | 65 | Birch high, ash weak to |
|  |  | End: 3:00 pm | 19.5 | 6.1 | O | 995.8 | 44 | moderate |
